# Frequency of pediatric HIV infection among high-risk children admitted to a tertiary care hospital at Sukkur, Sindh, Pakistan

**DOI:** 10.1186/s12879-025-11256-z

**Published:** 2025-07-01

**Authors:** Waqar Ahmed, Israr Ahmed, Fareeda Bhanbhro, Asghar Kerio, Arbab Ali, Sajid Ali, Parveen Wassan

**Affiliations:** 1 Sindh institute of child health and neonatology (SICHN), Sukkur, Pakistan; 2 Pir Abdul Qadir Shah Jeelani Institute of Medical Sciences, Gambat, Pakistan

**Keywords:** Pediatric HIV, High-Risk children, HIV screening, Blood transfusion, Unsafe needle practices

## Abstract

**Background:**

Pediatric HIV remains a significant public health concern, particularly in resource-limited settings such as Pakistan. Early diagnosis and intervention are essential for improving outcomes. This study aimed to determine the frequency of pediatric HIV infections among high-risk children admitted to a tertiary care hospital in Sukkur, Sindh.

**Study design:**

Retrospective cross-sectional study.

**Methodology:**

A total of 125 high-risk children were screened for HIV via the HIV 1/2 rapid test cassette. Blood samples were collected, processed, and tested following standard safety protocols. Only screening tests were performed, and confirmatory tests such as PCR, CD4 counts, and genetic workups were not available.

**Results:**

Among 125 children, 12 (9.6%) tested HIV-positive. A male predominance was observed (58.3% male, 41.7% female). Three-fourths of HIV-positive cases were from Sindh, and one-fourth were from Balochistan. 50% had a history of unsafe injection practices, and 41.7% had a history of blood transfusion. None of the HIV-positive children had HIV-positive parents. Two HIV-positive children died during the study period. All tested negative for hepatitis B and C; 16.7% had tuberculosis co-infection. Only 33.3% of HIV-positive children were fully vaccinated.

**Conclusion:**

This study highlights the prevalence of pediatric HIV in Sukkur, emphasizing the need for enhanced screening, confirmatory diagnostics, and preventive strategies to curb transmission. The absence of advanced diagnostic facilities limits comprehensive disease evaluation and management.

## Introduction

Human immunodeficiency virus (HIV) is a retrovirus belonging to the Lentivirus genus. It primarily infects CD4 + T lymphocytes, leading to progressive immune system dysfunction [1]. If untreated, the infection can progress to acquired immunodeficiency syndrome (AIDS), which is characterized by profound immunosuppression and increased susceptibility to opportunistic infections and death [2].

HIV is transmitted through contact with infected body fluids, including blood, semen, vaginal secretions, rectal fluids, and breast milk. Common routes include unprotected sexual intercourse, mother-to-child transmission during pregnancy, childbirth, or breastfeeding, and exposure to contaminated medical equipment or unscreened blood products [3].

Globally, HIV/AIDS remains a major public health challenge, with approximately 37.9 million people currently living with the virus, including 1.8 million children under the age of 15 [3–7]. AIDS is one of the leading causes of death worldwide, with over 34 million deaths recorded to date, and 96% of affected individuals residing in low- and middle-income countries [4].

In Pakistan, the burden of HIV has been steadily increasing. As of 2018, an estimated 160,000 individuals were living with HIV, including 2.2% children under 15 years [8]. According to more recent WHO data, around 94,000 people in Pakistan—including 2,100 women and children—are currently affected, with approximately 2,800 AIDS-related deaths annually [3, 8–11]. The country’s HIV-related mortality rate (1.5 per 100,000) exceeds that of many neighboring nations, reflecting substantial gaps in early detection, treatment access, and infection control. Sindh province bears the highest burden of HIV/AIDS in Pakistan, particularly among pediatric populations [4]. A critical example is the 2019 outbreak in Larkana, where more than 700 children were diagnosed with HIV—primarily attributed to unsafe injection practices, syringe reuse, and inadequate regulation of healthcare providers [4–6, 9]. The outbreak received both national and international media attention, triggering widespread concern about infection control practices and prompting expanded HIV screening campaigns and calls for systemic healthcare reform in Pakistan. It served as a national alarm, exposing critical gaps in infection prevention and underscoring the vulnerability of children to healthcare-associated transmission.

These concerns highlight the need for region-specific data and focused research. This study aims to provide insight into healthcare-associated transmission risks, unsafe medical practices, and highlight the need for improved critical care and equitable allocation of HIV-related resources, including diagnostics, antiretroviral therapy, and trained staff.

## Materials and mehtods

This retrospective cross-sectional study aimed to determine the prevalence of HIV infections among high-risk pediatric patients at Children’s Hospital Sukkur. High-risk children were defined as those presenting with one or more of the following criteria: failure to thrive, recurrent infections, unexplained weight loss, chronic diarrhea, history of blood transfusion, or suspected unsafe injection practices. During the study period, HIV screening was performed only for a sub-set of admitted children who fulfilled these high-risk criteria, as universal screening was not feasible due to limited diagnostic resources. Data were collected from September 1, 2024, to December 31, 2024. Given the retrospective design of the study, no prior sample size calculation was performed. All available high-risk pediatric cases during the study period were included. Data were entered and analyzed using SPSS version 22.0 for descriptive statistics and tabulation. Parental HIV status was obtained from documented rapid test results recorded in hospital files.

### HIV screening method

HIV screening was performed using the Bioline HIV 1/2 3.0 Rapid diagnostic Cassette (Abbott Diagnostics Korea Inc), a lateral flow chromatographic immunoassay that detects antibodies to HIV-1 and HIV-2. The test is based on the double-antigen sandwich principle. The membrane is pre-coated with recombinant HIV antigens; if HIV-specific antibodies are present in the patient’s serum, they bind to the antigen–gold conjugate and migrate via capillary action to form visible lines at the test and control regions.

Blood samples were collected in yellow-top gel tubes and processed under biohazard safety protocols. After centrifugation, serum samples were applied to the test cassette along with a buffer solution. Each test was performed according to the manufacturer’s instructions, and results were interpreted within 15 min.

A result was considered positive if two colored lines appeared—one at the control (C) line and one at the test (T) line. A negative result was defined by the presence of only the control line, with no visible test line. If the control line did not appear, the result was deemed invalid, and the test was repeated using a new cassette.

All results were interpreted by trained laboratory personnel and documented in the patient’s clinical file. No confirmatory PCR or ELISA testing was available on site during the study period. According to the manufacturer, the test has a reported sensitivity of 99.5% and specificity of 99.8%.

### Ethical approval

Ethical approval was obtained from the Institutional Review Board of Children’s Hospital Sukkur. All patient data were anonymized. There was no direct patient contact.

## Results

Among 125 high-risk children screened, 12 (9.6%; 95% CI: 5.1%–16.1%) tested positive for HIV, while 113 (90.4%; 95% CI: 85.3%–95.6%) tested negative. Among the 12 positive patients 7 (58.3%) were males and 5 (41.7 %) were females as shown in Table 1. Almost all patients were from Baluchistan and Sindh provinces. Of the 12 HIV-positive cases, 9 (75%) were from Sindh province, including 3 from Khairpur, 2 from Ghotki, and one each from Sukkur, Shikarpur, and Kashmor. The remaining 3 cases (25%) were from Balochistan, with one each from Nasirabad, Jafferabad, and Osta Mohammad as shown in Table 2. These districts in southeastern Balochistan border upper Sindh, indicating a possible regional clustering of cases. Almost all positive patients were below 3^rd^ centile with respect to weight for age (Table 3). Failure to thrive is the most common indication for testing for human immunodeficiency virus (HIV). Other indications were weight loss and chronic diarrhea as shown in Table 4. Among the 12 patients who tested positive for human immunodeficiency virus (HIV), 5 (41.7%) had a history of blood transfusion. Needle stick/unsafe injection practices were found in 6 (50%) patients out of 12. All patients who tested positive for human immunodeficiency virus (HIV) in their parents, i.e. mothers and fathers were also tested and all were found to be negative, as shown in Table 5. The routine EPI vaccination status was evaluated, and 4 (33.3%) out 12 were fully vaccinated according to the schedule (Table 6). All patients who were positive for human immunodeficiency virus (HIV) were also tested for hepatitis B and C virus and were found to be negative as shown in Table 7. HIV patients were also tested for tuberculosis 2 patients (16.7%) out of 12 tested positive for tuberculosis. Two HIV-positive patients died during the study period, highlighting the severity of the disease and the need for urgent medical interventions. As shown in Table 8, most patients (66.6%) were diagnosed at Stage III, followed by 16.6% at Stage IV. Early-stage diagnosis was less frequent, with 8.33% each at Stage I and II, indicating a trend of late HIV presentation.

**Table 1:** Total number of patient record reviewed in the study (*n*=125)

**Total Patients**	***n*** **=125**
HIV Positive	12 (9.6%)
HIV Negative	113 (90.4%)
Male	07 (58.3%)
Female	05 (41.7%)

**Table 2:** District-wise distribution of HIV patients

District	**Province**	**Frequency **
Jafferabad	Balochistan	1 (8.3%)
Kashmore	Sindh	2 (16.7%)
Khairpur	Sindh	3 (25%)
Nasirabad	Balochistan	1 (8.3%)
Osta Mohammad	Balochistan	1 (8.3%)
Shikarpur	Sindh	1 (8.3%)
Sukkur	Sindh	1 (8.3%)
Ghotki	Sindh	2 (16.7%)

**Table 3:** Age-related weight patterns among HIV cases

BELOW 3RD CENTILE	12 (100%)

**Table 4:** Clinical and epidemiological indications for HIV testing

Indications of HIV testing
Failure to thrive
Blood transfusion
Unsafe needle practice
Recurrent fever
Loss of weight
Recurrent Diarhea
Recurrent chest infectioins
Family history of HIV
Persistent lypmhadenopathy
Persistent thrombocytopenia
Seborrheic dermatitis

**Table 5:** Common risk factors associated with HIV positivity

Risk Factors	Present	Absent
History of blood transfusion.	5 (41.7%)	7 (58.3%)
History of unsafe needle practice	6 (50%)	6 (50%)
Mother HIV status.	0 (0%)	12 (100%)
Father HIV status.	0 (0%)	12 (100%)

**Table 6:** Vaccination status (routine EPI) among HIV positive children

Vaccinated	Not vaccinated
4 (33.3%)	8 (66.7%)

**Table 7:** Co-infection status (HBV, HCV, TB) in HIV-positive children

	Hepatitis B	Hepatitis C	TB
Positive	0 (0%)	0 (0%)	2 (16.7%)
Negative	12 (100%)	12 (100%)	10 (83.3%)

**Table 8:** WHO staging of HIV at time of diagnosis

**Stage**	Frequency
I	1(8.33%)
II	1(8.33%)
III	8(66.6%)
IV	2(16.6%)

## Discussion

This study highlights a concerning prevalence of pediatric HIV among high-risk children admitted to a tertiary care hospital in Sukkur, Sindh. The HIV positivity rate of 9.6% observed in our study is significantly higher than national estimates, which suggest that approximately 2.2% of total HIV cases in Pakistan occur in children under 15 years of age [1]. A striking finding is that none of the HIV-positive children had parents who tested HIV-positive, strongly suggesting a non-vertical (horizontal) route of transmission. Globally, vertical transmission remains the predominant mode, accounting for over 90% of pediatric HIV infections according to UNAIDS and WHO [12]. In contrast, 50% of our cohort had a history of unsafe injection practices and 41.7% had received blood transfusions—indicating possible iatrogenic transmission. This pattern is consistent with the 2019 Larkana outbreak, where most HIV-positive children had HIV-negative mothers and shared histories of repeated injections with unsafe equipment [7, 13].

The gender distribution in our sample showed a slight male predominance (58.3%), consistent with some international data, although no biological rationale is firmly established. This may reflect healthcare-seeking behavior or sampling variation due to the small sample size [12, 14–16]. Geographically, most HIV-positive children were from Sindh (75%)—notably Khairpur, Kashmor, Ghotki, and Sukkur—while the remaining 25% were from adjacent districts in Balochistan. These areas share common healthcare challenges: poor immunization coverage, inadequate infection control, and widespread use of informal healthcare services, all of which may contribute to the transmission. This distribution reinforces earlier reports that Sindh carries the highest burden of HIV/AIDS in Pakistan [6].

Clinically, failure to thrive, weight loss, and chronic diarrhea were prominent features, aligning with classical pediatric HIV presentations. It is also concerning that only 33.3% of HIV-positive children were fully vaccinated, increasing their risk of preventable opportunistic infections [6–11].

These findings highlight the urgent need for broader HIV screening criteria in pediatric populations, extending beyond children of HIV-positive mothers. The absence of vertical transmission and the strong association with unsafe medical practices call for immediate public health action, including improved infection control, stricter regulation of medical procedures, and safer transfusion protocols.

Tuberculosis co-infection was found in 16.7% of cases—slightly lower than Pakistan’s national estimate of 23% [11]. None of the children tested positive for hepatitis B or C, which differs from findings in adult HIV cohorts. This points to a localized pattern of pediatric HIV transmission, primarily driven by unsafe healthcare practices rather than maternal transmission. Efforts were made to trace all HIV-positive children identified during the study. The corresponding author personally contacted caregivers using mobile numbers from hospital records. One patient had died, and two were successfully referred to the HIV Treatment Center in Larkana for antiretroviral therapy. The remaining families, however, did not follow through with care due to transportation barriers, financial constraints, and stigma. In response, hospital administration has been notified of the HIV burden, and protocols for screening high-risk admissions have been formalized. A formal request has also been submitted to the Sindh AIDS Control Program to establish a dedicated HIV treatment unit in Sukkur, aiming to reduce reliance on referral centers in distant districts.

These findings call for immediate, multi-level interventions. Routine HIV screening should be expanded to include all high-risk pediatric admissions. Infection prevention practices must be reinforced across healthcare facilities. Public education campaigns should target early testing and reduction of stigma. Immunization efforts must be scaled up for vulnerable children. Finally, it is essential to address broader social determinants—poverty, health literacy, and care accessibility—to reduce the pediatric HIV burden in this region.

## Conclusion

This study found a high HIV positivity rate of 9.6% among high-risk pediatric patients admitted to a tertiary care hospital in Sukkur, Sindh — substantially exceeding national estimates. The majority of HIV-positive cases were below five years of age, with a male predominance and significant associations with unsafe injection practices (50%) and blood transfusions (41.7%). None of the HIV-positive children had HIV-positive parents, supporting horizontal rather than vertical transmission. These findings highlight the urgent need for expanded screening, healthcare worker training, safe injection practices, and robust pediatric HIV surveillance in high-risk regions of Pakistan.

## Data Availability

The datasets generated and/or analyzed during the current study are available from the corresponding author on reasonable request.

## References

[1] Luzuriaga K, Lynne M, Mofenson. Challenges in the elimination of pediatric HIV-1. Infection. New Engl J Med Vol. 2016;374(8):761–70. 10.1056/NEJMra1505256. 10.1056/NEJMra1505256 26933850

[2] van Heuvel Y, Schatz S, Rosengarten JF, Stitz J, Infectious RNA. Human immunodeficiency virus (HIV) biology, therapeutic intervention, and the quest for a vaccine. Toxins (Basel). 2022;14(2):138. 10.3390/toxins14020138. PMID: 35202165; PMCID: PMC8876946. 35202165 10.3390/toxins14020138 PMC8876946

[3] Meissner ME, Talledge N, Mansky LM. Molecular biology and diversification of human retroviruses. Front Virol. 2022;2:872599. 10.3389/fviro.2022.872599. Epub 2022 Jun 2. PMID: 35783361; PMCID: PMC9242851. 35783361 10.3389/fviro.2022.872599 PMC9242851

[4] Chuadhry MA, Nawaz SM, Zafar S. National efforts to combat HIV/AIDS in pakistan: an analysis from prevalence to prevention. Webology. 2021;18(4).

[5] World health Organization (WHO). [Online] 2014. Available from: URL: http://www.who.int/hiv/en/. Cited 2019 April 24.

[6] Joulaei H, Shooshtarian S, Dianatinasab M. Is UNAIDS 90-90-90 target a dream or a reality for middle East and North Africa region on ending the AIDS epidemic?? A review study. AIDS Rev. 2018;20(2). 10.24875/AIDSRev.M18000020 29938702

[7] Mir F, Mahmood F, Siddiqui AR, Baqi S, Abidi SH, Kazi AM, Nathwani AA, Ladhani A, Qamar FN, Soofi SB, Memon SA. HIV infection predominantly affecting children in sindh, pakistan, 2019: a cross-sectional study of an outbreak. Lancet Infect Dis. 2020;20(3):362–70. 31866326 10.1016/S1473-3099(19)30743-1

[8] Siddiqui AR, Nathwani AA, Abidi SH, Mahmood SF, Azam I, Sawani S, Kazi AM, Hotwani A, Memon SA, Soomro J, Shaikh SA. Investigation of an extensive outbreak of HIV infection among children in sindh, pakistan: protocol for a matched case–control study. BMJ Open. 2020;10(3):e036723. 32213527 10.1136/bmjopen-2019-036723 PMC7170612

[9] Mehnaz A. Emerging threat of HIV/AIDS in children in Pakistan. J Pak Med Assoc. 2020;70(8):1312–3. 32794477

[10] Letendre SL, McCutchan JA, Childers ME, Woods SP, Lazzaretto D, Heaton RK, Grant I, Ellis RJ, HNRC Group. Enhancing antiretroviral therapy for human immunodeficiency virus cognitive disorders. Ann Neurol. 2004;56(3):416– 23. 10.1002/ana.20198. PMID: 15349869. 10.1002/ana.20198 15349869

[11] Emmanuel F, Blanchard J, Hasan Z. The HIV/AIDS surveillance project mapping approach for mapping and size Estimation for groups at high risk of HIV in Pakistan. AIDS. 2010;24:77–84. 20610953 10.1097/01.aids.0000386737.25296.c4

[12] Global report: UNAIDS report on the global AIDS epidemic. 2012. [Online]. Available from: URL: http://www.unaids.org/en/media/unaids/contentassets/documents/epidemiology/gr2012/20121120_UNAIDS_Global_Report_2012_en.pdf. Cited 2013 Sept 18.

[13] Murray CJ, Ortblad KF, Guinovart C, Lim SS, Wolock TM, Roberts DA, et al. Global, regional and National incidence and mortality for HIV, tuberculosis and malaria during 1990–2013: a systemic analysis for the global burden of disease study. Lancet. 2013;384:1005–70. 10.1016/S0140-6736(14)60844-8 PMC4202387 25059949

[14] Treatment and PPT centers National Aids Control programmer (NACP). [Online]. Available from: URL: www.nacp.gov.pk. Cited 2016 June 09.

[15] Melis T, Sahle T, Haile K, Timerga A, Zewdie A, Wegu Y, Zepire K, Bedewi J. Providing anti-retroviral treatment did not achieve the ambition of ‘joint united nations program on HIV/AIDS (UNAIDS) among HIV positive patient in ethiopia’: a systematic review and meta-analysis. J Pharm Policy Pract. 2024;17(1):2290672. 38234997 10.1080/20523211.2023.2290672 PMC10793635

[16] Hlophe LD, Tamuzi JL, Shumba C, Nyasulu PS. Barriers to anti-retroviral therapy adherence among adolescents aged 10 to 19 years living with HIV in sub-Saharan africa: A mixed-methods systematic review protocol. PLoS ONE. 2022;17(9):e0273435. 36178934 10.1371/journal.pone.0273435 PMC9524658

